# Health literacy and risk behaviours related to sexually transmitted infections among Portuguese university students: a cross-sectional study

**DOI:** 10.1186/s12889-025-25904-1

**Published:** 2026-01-14

**Authors:** Beatriz Quaresma, Paulo Santos

**Affiliations:** 1 Faculty of Medicine, University of Porto, Porto, Portugal; 2 RISE-health@RISE, MEDCIDS, Faculty of Medicine, University of Porto, Porto, Portugal

**Keywords:** Sexually transmitted infections, Young adults, Risk behaviour, Sex education, Health literacy

## Abstract

**Background:**

Sexually transmitted infections (STIs) represent a growing concern for public health worldwide, particularly following the COVID-19 pandemic. Adolescents and young adults are disproportionately affected. Factual knowledge and personal beliefs seem to play a role in preventing sexual risk behaviours. Furthermore, adequate health literacy has been shown to improve health outcomes. Understanding the determinants of safer sexual practices is essential for developing more effective sexual health education interventions. Accordingly, this study aims to assess knowledge, attitudes, and practices towards STIs and health literacy levels in Portuguese young adults.

**Methods:**

A cross-sectional study was conducted among students at the University of Porto from September 2024 to January 2025. An online, structured, and anonymised questionnaire focused on STI aetiology, modes of transmission, clinical aspects, sexual risk behaviours, preventive practices, sociodemographic characteristics, and health literacy. Data were analysed using both descriptive and inferential methods. Lastly, the Mann-Whitney U test, Kruskal-Wallis H test, and multiple linear regression were used.

**Results:**

Among 790 participants (median age 22.0 years), 84.0% had already initiated their sexual activity. Around half (49.7%) had problematic health literacy levels. HIV infection, genital herpes, and hepatitis B were the most widely known infections. Meanwhile, 50.9% were unaware of trichomoniasis, and 65.8% did not recognise parasites as potential etiological agents for STIs. Half of them expressed concern about contracting an STI, and similar proportions had used condoms at their last sexual intercourse (47.7%) and had been tested for HIV (51.5%). Regression models revealed that work experience in healthcare and better health literacy predict higher knowledge of STIs. In turn, knowledge was the strongest predictor of better attitudes. Moreover, being female, currently in a relationship, and having had a sexual debut at an older age were associated with safer sexual practices.

**Conclusions:**

This study identifies relevant knowledge gaps, determinants of sexual risk behaviours, and insufficient health literacy levels in Portuguese university students. These findings reinforce the need for accessible, proactive sexual health education interventions capable of empowering individuals and that are tailored to the values and beliefs of today’s youth.

**Supplementary Information:**

The online version contains supplementary material available at 10.1186/s12889-025-25904-1.

## Introduction

Sexually transmitted infections (STIs) represent a growing public health challenge worldwide. It is estimated that one million new cases of curable STIs are acquired globally every day. However, this number is likely being underestimated due to underreporting, asymptomatic infections, and social stigma [1]. These infections are transmitted mainly via sexual intercourse and are caused by over 30 different bacteria, viruses, and parasites. STIs adversely impact sexual and reproductive health, potentially leading to infertility, inflammatory pelvic disease, obstetric complications, congenital disabilities, and even death. They also increase susceptibility to Human immunodeficiency virus/Acquired immunodeficiency syndrome (HIV/AIDS) infection [1].

The Centres for Disease Control and Prevention (CDC) confirmed an increasing trend in transmission rates, achieving 2.5 million reported cases of chlamydia, gonorrhoea, and syphilis in the United States (US) in the year 2022. Nearly half (49,8%) of these new cases were among teenagers and young adults aged 15–24 years, who represented only a quarter of the sexually experienced population [2]. A similar trend is also occurring in Europe. In 2023, we saw an increase in new cases of gonorrhoea by 31%, 13% for syphilis, and 3% for chlamydia compared to the previous year [3].

Chlamydia remains the most prevalent bacterial STI worldwide and is often asymptomatic. In Portugal, incidence rates increased from 8.9 to 13.4 cases per 100,000 inhabitants between 2021 and 2023, with a male-to-female ratio of 2.3, one of the highest in the European Union (EU) and the European Economic Area (EEA) [4].

In 2023, gonorrhoea exhibited the most considerable rise in new cases among STIs across the EU/EEA, reaching the highest annual incidence since surveillance began in 2009, which raises concerns about its high incidence, potential health complications, and the challenges facing its antimicrobial resistance [5]. Between mid-2022 and early 2023, a gonorrhoea outbreak was observed among at least 15 EU/EEA countries, affecting all age groups and sexes, particularly individuals aged 20–24 years. This period followed the easing of COVID-19 restrictions, which had previously delayed sexual initiation. The subsequent increase in social contact may have led to riskier sexual behaviours [6]. Portugal experienced a 97.3% increase in new cases since 2019 [7].

The EU incidence of HIV infection decreased by 15.9% from 2014 to 2023, with a slight increase of 11.8% between 2022 and 2023. Approximately 52.7% of these cases were diagnosed late, defined as a CD4 cell count below 350 cells/mm³ [8]. Late diagnosis is linked to inadequate HIV testing, which is thought to be due to insufficient knowledge regarding HIV transmission, symptoms, and signs [9].

Additionally, social and cultural stigma and self-stigma associated with STIs remain high, significantly impacting individuals’ mental health, their demand for health care, and adherence to preventive strategies, screening, and treatment. At the same time, it poses barriers, hindering the implementation of prevention and educational programs and control policies in society, which leads to insufficient sexual health education and low sexual health literacy [10–12].

Health literacy (HL) is defined as the degree to which individuals can “access, understand, appraise and use information and services in ways that promote and maintain good health and well-being” [13]. Higher levels of health literacy are sought to contribute to better health outcomes, including on STIs, by enhancing understanding of disease etiologic mechanisms, transmission, and primary prevention; recognition of signs and symptoms, informed decision-making, such as adherence to barrier methods and screening programs; appropriate healthcare-seeking behaviours; and compliance with the treatments. Several studies have linked higher HL to increased knowledge of HIV-related disease and better adherence to antiretroviral therapy [14, 15]. Other research found positive associations between health literacy, knowledge about Human papillomavirus (HPV), HPV vaccine awareness [16, 17], and vaccination rates [18]. However, the relationship between HL and sexual health outcomes is not entirely understood, requiring further research [19]. Apart from this relation of HL to HPV and HIV, the literature is scarce regarding its impact on a broader range of STIs.

This study is conducted in the context of a global resurgence of STIs following the COVID-19 pandemic, alongside notable changes in sexual behaviours and patterns. These shifts, especially among young people, include a decline in condom use, increased substance use before sex, and a rising prevalence of multiple sexual partners [20, 21]. Furthermore, previous research suggests that limited STI knowledge among young adults, including university students, may increase their susceptibility to engage in risky sexual behaviours [22, 23].

Considering this, our study aims primarily to characterise knowledge, attitudes, and practices (KAP) related to STIs among college students. Specifically, the objectives are: (1) to identify current patterns of sexual behaviours in this population; (2) to explore predictors of unhealthy sexual behaviours based on sociodemographic, financial, and educational characteristics; and (3) to examine the relationship between KAP and levels of health literacy. To achieve these aims, we employed previously validated instruments: the KAP questionnaire, originally developed and validated by Folasayo et al. and previously used among college students in similar academic contexts [24]; and the validated Portuguese version of the European Health Literacy Survey Questionnaire (HLS-EU-Q12) by Arriaga et al. and previously applied to a Portuguese population aged 16 and older [25].

## Materials and methods

This cross-sectional observational study recruited active students from the University of Porto (UP) in 2024/2025. All students were eligible to participate without any limitations. The UP currently has 31,243 students, distributed across 14 faculties, with the majority aged 17–24 years, although a few are older. As one of the country’s leading academic institutions, UP attracts students from diverse regions and socio-economic backgrounds, making it a representative sample of national students with access to higher education. Furthermore, the Porto Metropolitan area is a densely populated region with consistently high STI notification rates [26].

We developed an online, self-administered, and anonymised questionnaire using Google Forms. Invitations were shared via institutional email to potentially all the students in the UP by the central services of the University and reinforced using student associations’ diffusion and social media invitations upon authorisation of UP’s Data Protection Unit. Recruitment and data collection took place from September 10, 2024, to January 31, 2025. No follow-up was needed.

The questionnaire was restricted to one response per email, using a Google^®^ unique login system. Informed consent was provided on the first page, and the intention to participate was assessed using the first question. A disagreement led to an immediate dropout. To minimise social desirability bias, the questionnaire was anonymous, with neutral phrasing of questions to avoid leading responses. Data privacy and protection were secured in accordance with the General Data Protection Regulation (GDPR). The data were stored in the servers of the UP and remained accessible for 5 years.

The questionnaire comprised 95 questions, divided into seven sections, including open-ended, closed-ended, multiple-choice, multiple-answer, and 5-point Likert Scale options (Supplement 1).

The first three sections, Knowledge, Attitudes, and Practices, were based on a previously validated questionnaire also applied to college students by Folasayo et al. [24], following re-use authorisation. Our team meticulously translated the questionnaire into Portuguese. The questionnaire underwent a simplified pilot test to assess and improve its comprehensibility before distribution, with minor adjustments made to better align with the reality of our students. A formal validation of the final version was not performed.

The section “Knowledge” entailed seven questions, focusing on aspects such as aetiology, transmission routes, symptoms, complications of STIs, contraception, and risky behaviours. The response method through a 5-point Likert Scale of Agreement (1 = “totally disagree” to 5 = “totally agree”) or of Knowledge (1 = “none” to 5 = “profound”) and the multiple answer format for the other two questions, instead of the yes/no, as in the original questionnaire, was used. This decision enabled greater freedom and response precision in questions that required a range of knowledge [27]. An option “neither agree nor disagree” was included to address those who could not make a clear judgment or stand for a genuine neutral opinion, avoiding forced and thus unreliable/inaccurate responses. A knowledge score was calculated as the mean of all answered questions, using Likert Scale points (1–5) and assigning five points for each correct multiple-choice answer. The mean score was then scaled proportionally to 100, resulting in a knowledge score range of 14,1 to 100%. The same procedure was applied to the sections on attitudes (range: 20%−100%) and practice (range: 12,9%−100%). Participants who pointed out the answers “I do not know” or “I do not want to answer” in more than 20% of each dimension were excluded and treated as non-responders for that component.

The section “Attitudes” contained 18 questions assessing perception towards STIs, whose response format followed the Likert Scale of Agreement, and one ranking question related to personal concerns about unprotected sexual intercourse. The item “I am worried about contracting STIs” was excluded from the composite score due to high response variance and concerns about interpretive ambiguity.

The “Practices” included nine questions, four evaluating condom use, age of sexual debut, HIV testing, and sexual partners, and five related to the frequency of risky behaviours. Within this section, some questions were based on an adapted 5-point Likert Scale of Frequency (1= “never” to 5 = “very often”). To minimise response bias and reduce the risk of inaccurate data, we included the option “doesn’t apply”. To the original question, a quantity of drinking alcohol was added to obtain a more accurate reply. The question “Do you read pornographic materials?” was adapted to “Do you read/watch pornographic materials?” to adjust to our population’s expected habits. The question regarding partners in the last 12 months was altered from a yes/no answer to a multiple-choice question for a more comprehensive analysis. The question “Do you have sex with commercial sex workers?” was rephrased to assess practices either as a receptor or a provider.

The section of Sexual History included questions such as the age of first sexual intercourse, whether a condom was used, and the current contraception methods. Sexual intercourse was defined throughout the questionnaire as a practice that involves both sexual organs and a second person, encompassing vaginal, anal, and oral sex.

Sexual Orientation was described by the Kinsey Scale [28–30]. This scale varies from 0 to 6, with 0 (exclusively heterosexual) and 6 (exclusively homosexual). The range between 2 and 4 represents the spectrum of bisexual behaviour, with 3 being the equal attraction to both sexes [30, 31]. A total of seven questions regarding sexual intercourse preferences, fantasies, emotional bonds, and attraction were employed, and answers varied on a scale from 1 to 5 (1= “only with the opposite sex” to 5= “only with the same sex”). The obtained mean scores were converted to the final scale of 0–6 and then rounded to fit within each category of the Kinsey Scale. As with KAP scores, more than 20% of “I do not know” or “I do not want to answer” answers resulted in treating the participant as a non-respondent.

Furthermore, the section on Health Literacy comprised the HLS-EU-Q12 2018 by Finbråten et al., a short, validated version with good reliability [32] of the European Health Literacy Survey Questionnaire (HLS-EU-Q47), which was reused with authorisation. The HLS19 Instrument used in this research was developed within the context of “HLS19 – the International Health Literacy Population Survey 2019–2021” of the WHO Action Network on Measuring Population and Organisational Health Literacy (M-POHL). We used the Portuguese translation by Arriaga et al. of the 12-item HLS-EU [25], allowing us to calculate the score of HL, following the equation (index = (mean − 1) * 50/3), ranging from 0 to 50 points. The levels of HL were classified as excellent (43–50), sufficient (34–42), problematic (26–33), and inadequate (0–25). The HL score was not calculated for participants with an “I don’t know” response rate exceeding 20% of the total questions, in accordance with the recommended methodology of HLS-EU [33, 34]. This exclusion criterion was applied to all sections of the questionnaire.

The resumed inquiry about sociodemographic variables was partially based on the sociodemographic characterisation from the HLS-EU-Q47. The variables used were biological sex, age, faculty, former education in healthcare, parents’ nationality, marital status, living arrangements, work situation, and socioeconomic self-evaluation. Social status was characterised through a 10-point scale, with low (1–3), middle (4–7), and high class (8–10) [35]. The minimum sample size was established at 765 participants, calculated for a 95% confidence level, a 3.5% margin of error, and an unknown population distribution [36].

Data analysis included frequencies and proportions for categorical variables, as well as medians, means, and standard deviations (SDs) for continuous variables. These results were presented in tables and bar charts, along with 95% confidence intervals (95% CI). For the construction of the figures, the 5-point Likert Scale was converted to a correct/incorrect answer, with “agree” and “totally agree” considered correct when pointing towards the right answer. The same approach was applied to the Likert Scales for risky behaviours, where “never”, “rarely”, and “occasionally” were considered adequate responses. The Mann-Whitney U Test examined differences between dichotomic variables, KAP scores, and HL. The Kruskal-Wallis H Test was performed to compare more than two groups. The normality of the data was assessed using the Kolmogorov–Smirnov test. The covariates with statistical significance in relation to the outcomes, primarily assessed through bivariate analysis, were introduced into multiple linear regression models using the Enter method. Three models were created, each using one of the following as a dependent variable: knowledge, attitudes, or practices. Therefore, we could examine associations between KAP scores and sociodemographic characteristics, including HL. Practice scores were not included in the first two models, as they reflected only the knowledge and attitudes of sexually active participants, thereby avoiding selection bias. Beta coefficients, 95% confidence intervals, and p-values were reported to quantify the strength, precision, and significance of the associations observed. The goodness of fit of linear regression was ascertained for multicollinearity, linearity, homoscedasticity, independence, and normality of residuals. The last was assessed through histogram representation and the Kolmogorov-Smirnov Test.

A p-value of 0.05 was set as the threshold for statistical significance.

No imputation methods were used for variables containing missing data.

Data were transferred to and registered in Microsoft Office Excel 2023 © and encoded and analysed through IBM SPSS Statistics ©, version 28 (IBM Corp., Armonk, N.Y., USA).

This study is part of the Project S4Sex, which aims to fill gaps in sexual education in Portugal by providing community-based primary prevention programs for a salubrious, safe, sensible, and satisfying sexuality. This project aligns with the prevention branch of research priorities for STIs, as published by the WHO for 2022–2030 [37].

This study adhered to the ethical principles outlined in the Declaration of Helsinki and the Convention of Oviedo. The protocol was appraised and accepted by the Faculty of Medicine Ethics Committee, UP (protocol code: 204/2024; date of approval: 27th June 2024).

## Results

### Demographic data

A total of 790 participants completed the survey and were considered valid. Most of the participants were females (68.9%), without training or education in healthcare (74.8%), not currently in a relationship (46.6%), middle-class (85.8%), and with at least one parent born in Portugal (88,6%). The mean age was 24.24 ± 7.01 (median 22.0). The participants came from all 14 faculties, with the highest contributions from the Faculty of Medicine (FMUP) at 22.4%, the Faculty of Sciences (FCUP) at 14.3%, and the Faculty of Engineering (FEUP) at 13.0%. Table 1 presents the sociodemographic characteristics of the sample and the mean health literacy scores for each subgroup within these variables.

**Table 1:** Sociodemographic Characteristics and respective mean Literacy Scores

	No. (%) *n*= 790	Mean Literacy Score (0–50) ± SD *n*= 753	*p*-value†
Biological Sex (*n* = 790)			0.232
Female	544 (68.9)	31.75±7.51	
Male	246 (31.1)	32.21±7.26	
Mean Age (y) (*n* = 789)	24.24 ± 7.10		
Age Groups (y) (*n* = 789)			0.025
17–21	312 (39.6)	30.93±6.87	
22–26	308 (39.0)	32.59 ± 7.49	
27–31	86 (10.9)	32.83 ± 8.80	
≥ 32	83 (10.5)	31.88 ± 7.42	
Parent’s nationality (*n* = 776)			0.330
At least one is Portuguese	685 (88.3)	31.91 ± 7.25	
At least one is from an EU-country but not from Portugal	18 (2.3)	34.66 ± 9.79	
Both parents were born outside of the EU	73 (9.4)	30.84 ± 8.55	
Marital Status (*n* = 779)			0.900
Single	686 (88.0)	31.91 ± 7.52	
Married or non-marital partnership	84 (10.8)	31.95 ± 6.90	
Separated or divorced	9 (1.2)	32.17 ± 7.99	
Widowed	0 (0)	-	
Living arrangements (*n* = 766)			0.973
Single/living alone	357 (46.6)	31.74 ± 7.40	
Cohabitation partnership	126 (16.5)	32.10 ± 6.74	
In a serious relationship, but not cohabiting	283 (36.9)	32.12 ± 7.91	
Have children? (*n* = 788)			0.594
Yes	48 (6.1)	31.48 ± 7.54	
No	740 (93.9)	31.93 ± 7.42	
Working Situation (*n* = 767)			0.388
Employed	227 (29.6)	32.10 ± 7.98	
Unemployed	37 (4.8)	30.02±6.57	
Student, unpaid work experience	503 (65.6)	31.98±7.26	
Previous training/work in healthcare, such as nursing, medicine, pharmacy (*n* = 779)			< 0.001
Yes	196 (25.2)	33.94 ± 7.99	
No	583 (74.8)	31.22 ± 7.16	
In the last 12 months, have you had difficulties paying for bills by the end of the month? (*n* = 677)			0.149**
Yes	153 (22.6)	31.50 ± 8.37	
No	524 (77.4)	32.38 ± 7.29	
Is it difficult for you to buy medication for yourself or your family? (*n* = 762)			< 0.001**
Yes	79 (10.4)	26.42 ± 7.38	
No	683 (89.6)	32.54 ± 7.22	
Is it difficult for you to have access to your assistant physician (considering time, health insurance, commuting costs, and transport)? (*n* = 755)			< 0.001**
Yes	275 (36.4)	29.83 ± 7.12	
No	480 (63.6)	33.14 ± 7.45	
Self-reported socioeconomic classification (*n* = 790)			0.271
Low class	24 (3.1)	30.19 ± 9.33	
Middle class	678 (85.8)	31.84 ± 7.31	
High class	88 (11.1)	32.73 ± 7.72	
Sexual Orientation (*n* = 759)			0.660
Heterosexual (0–1)	67 (8.8)	30.97 ± 8.34	
Bisexual (2–4)	638 (84.1)	32.00 ± 7.38	
Homosexual (5–6)	54 (7.1)	31.03 ± 6.66	
Mean age of sexual debut (y) (*n* = 655)	17.67 ± 2.53		
Age of sexual debut by groups (y) (*n* = 655)			0.059
12–15	118 (18.0)	33.42 ± 8.84	
16–21	491(75.0)	31.54 ± 7.20	
≥ 22	46 (7.0)	32.91 ± 7.79	
Did you use any contraceptive methods during your first sexual intercourse? (*n* = 626)*			0.134
Yes	521 (83.3)	32.27 ± 7.34	
No	101 (16.1)	30.50 ± 8.50	
I don’t know	4 (0.6)	32.64 ± 7.56	
Contraceptive Method at first sexual intercourse (*n* = 521)			0.297
Condom	478 (91.7)	32.32 ± 7.30	
Others	43 (8.3)	31.66 ± 7.89	
Usual contraceptive method (*n* = 665)			0.260
Condom	397 (59.7)	31.64 ± 7.50	
Others	268 (40.3)	32.39 ± 7.67	

The total number of participants decreased for some of the indicated variables due to the exclusion of responses marked as “I don’t know”

*Non-responses contributed to a reduced total number of valid cases

† Mann-Whitney test was applied to dichotomic variables. Kruskal-Wallis to the others

** The variables were dichotomised for analysis (most of the time or sometimes – “yes”; almost never or never – “no”) and (very difficult or difficult – “yes”; easy or very easy – “no”)

The majority (82.8%) reported already having had sexual intercourse, with a mean age for the first intercourse of 17.67 ± 2.53 years. Contraception was used in the first intercourse by 83.8% (91.7% used condoms, 35.8% birth-control pill, 3.3% coitus interruptus, 2.1% morning-after pill, 1.4% natural methods, like calendar, temperature, and cervical mucus, and 0.4% for both intrauterine device (IUD) and vaginal ring). In a regular contraception, students used condoms (59.5%) and contraceptive pills (43.4%).

### Health literacy (HL)

Almost half of the participants with a valid answer (*n* = 753) had a problematic level of health literacy (49.7%), 23.6% scored as sufficient, 17.4% scored as inadequate, and only 9.3% had an excellent level of health literacy. Higher levels of HL are associated with better access to medication (*p* < 0.001) and to the assistant physician (*p* < 0.001).

### Knowledge, Attitudes, and practices (KAP) scores on stis

#### Knowledge

The mean knowledge score was 73.54 ± 8.94 (*n* = 790). The self-perceived general knowledge of STIs, as assessed by a single question, was adequate in 50.9% of the students, as indicated by an answer on a 4–5 point Likert scale. As expected, the infection by HIV was the most well-known STI, with 74.0% of participants admitting an adequate to profound knowledge, followed by genital herpes and hepatitis B. The least known were granuloma inguinale, chancroid, and trichomoniasis. Table 2 summarises participants’ self-reported knowledge of STIs, both overall and for specific infections.

**Table 2:** Level of self-perceived Knowledge of STIs

Questions	“None” *n*(%)	“Minimum” *n*(%)	“Basic” *n*(%)	“Adequate” *n*(%)	“Profound” *n*(%)
All STIs (*n* = 790)	3 (0.4)	68 (8.6)	317 (40.1)	302 (38.2)	100 (12.7)
Gonorrohea (*n* = 787)*	71 (9.0)	187 (23.8)	245 (31.1)	239 (30.4)	45 (5.7)
Syphilis (*n* = 788)*	71 (9.0)	156 (19.8)	258 (32.7)	245 (31.1)	58 (7.4)
Chlamydia (*n* = 788)*	79 (10.0)	150 (19.0)	237 (30.1)	271 (34.4)	51 (6.5)
Chancroid (*n* = 772)*	409 (53.0)	116 (15.0)	147 (19.0)	85 (11.0)	15 (1.9)
Granuloma inguinale (*n* = 775)*	405 (52.3)	130 (16.8)	140 (18.1)	88 (11.4)	12 (1.5)
Genital Herpes (*n* = 788)*	28 (3.6)	114 (14.5)	227 (28.8)	318 (40.4)	101 (12.8)
HIV/AIDS (*n* = 790)	7 (0.9)	35 (4.4)	164 (20.8)	390 (49.4)	194 (24.6)
Hepatitis B (*n* = 789)*	63 (8.0)	141 (17.9)	217 (27.5)	273 (34.6)	95 (12.0)
Trichomoniasis (*n* = 770)*	392 (50.9)	120 (15.6)	107 (13.9)	120 (15.6)	31 (4.0)

*The total number of participants decreased for the indicated variables due to an “I don’t know” response

**Fig. 1:**
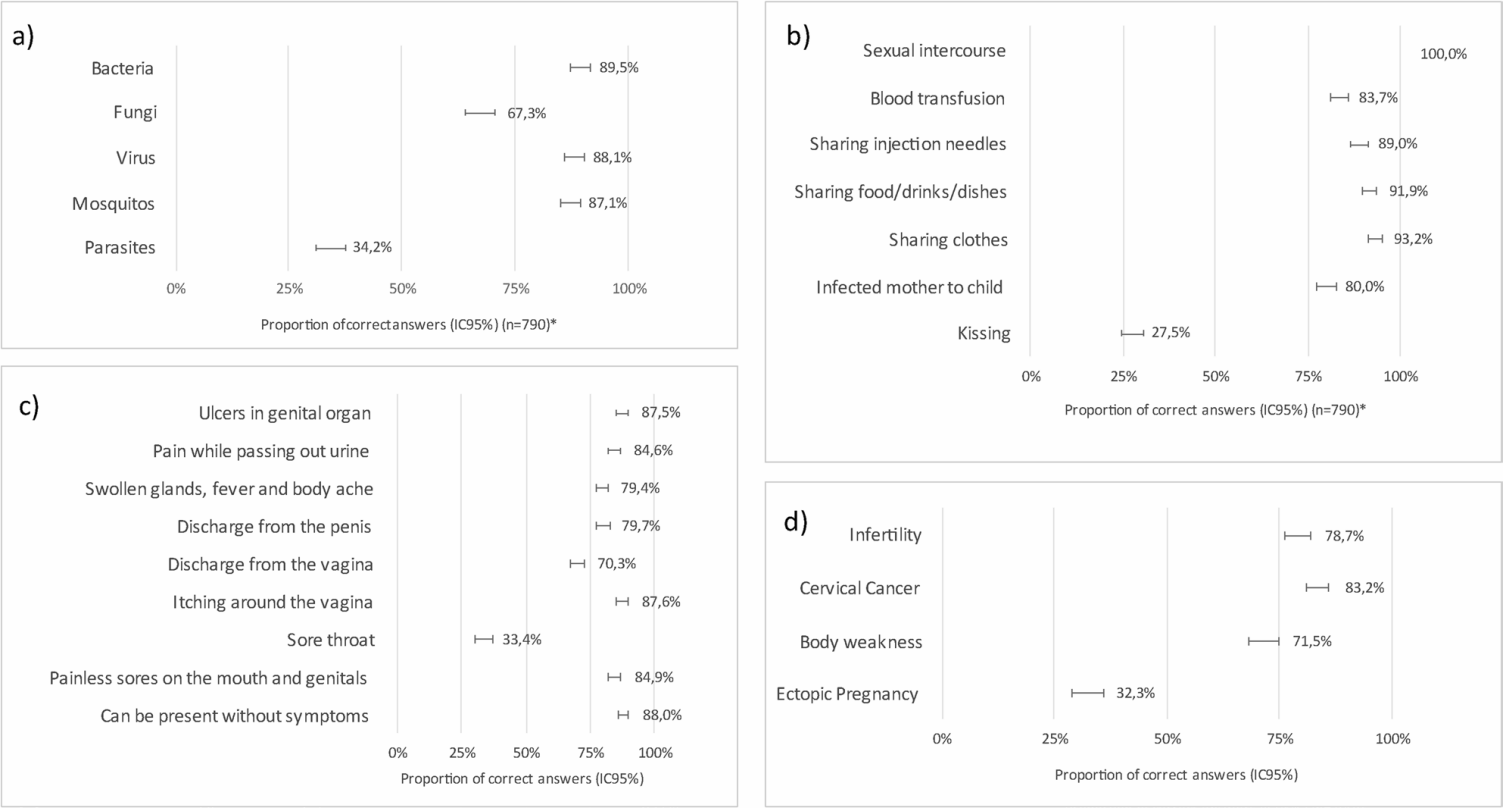
Knowledge on STIs (proportion of correct answers) **a**) Identification of aetiological agents; **b**) Identification of modes of transmission; **c**) Identification of possible signs and symptoms; **d**) Identification of complications associated with sexually transmitted infection

Figure 1 illustrates the proportion of correct answers to knowledge questions regarding aetiology, modes of transmission, signs and symptoms, and complications of STIs. Bacteria and viruses were correctly identified as etiological agents, while mosquitoes were correctly identified as not being etiological agents. However, 65.8% and 32.7% didn’t recognise parasites and fungi, respectively, as important pathogens. The symptoms and complications of STIs were recognised above 80%, except sore throat, where only 33.4% selected this option correctly, and ectopic pregnancy, which was the least recognised complication (by only 32.3%).

**Fig. 2:**
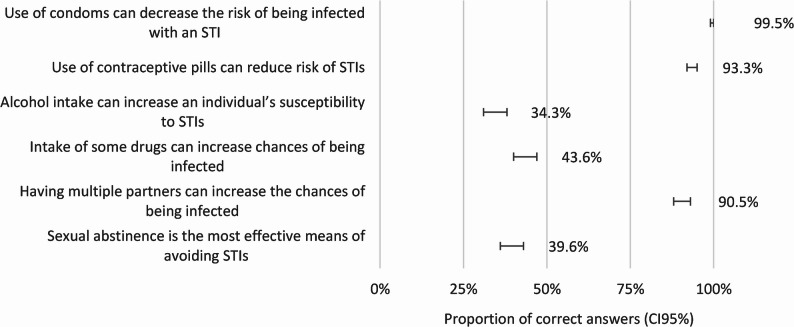
Knowledge on Preventive Practices and Risky Behaviours (proportion of correct answers)

More than 90% of participants demonstrated suitable knowledge regarding contraception and barrier methods (Fig. 2). When asked about sexual risk behaviours, 34.3% agreed that alcohol intake may increase STI transmission, and 43.6% correctly answered that some drugs are also associated with an increase in the risk.

Knowledge scores were significantly higher in women than men (73.99 ± 9.02 vs. 72.53±8.71; *p* = 0.04). Participants with health work-related experience scored higher than the others (81.10±7.94 vs. 71.01±7.82; *p* < 0.001), and participants who already had sex also scored significantly higher (74.01±8.79 vs. 71.01±7.19; *p* < 0.001). There were significant differences among age groups (*p* < 0.001). The participants aged 22–26 were the most knowledgeable (76.28 ± 9.27), and those aged 17–21 scored the lowest (70.03 ± 7.18). Participants who have had sexual intercourse exclusively with the opposite sex had lower knowledge scores than those who have had it, exclusively or not, with the same sex (73.50 ± 9.07 vs. 75.74 ± 8.06; *p* = 0.002).

Moreover, higher scores were observed among participants who reported greater ease in accessing and purchasing medications (69.95 ± 7.48 vs. 74.05 ± 9.00; *p* < 0.001), as well as those who reported easier access to their assistant physician (74.28 ± 9.02 vs. 72.48 ± 8.71; *p* = 0.02).

#### Attitudes

**Fig. 3:**
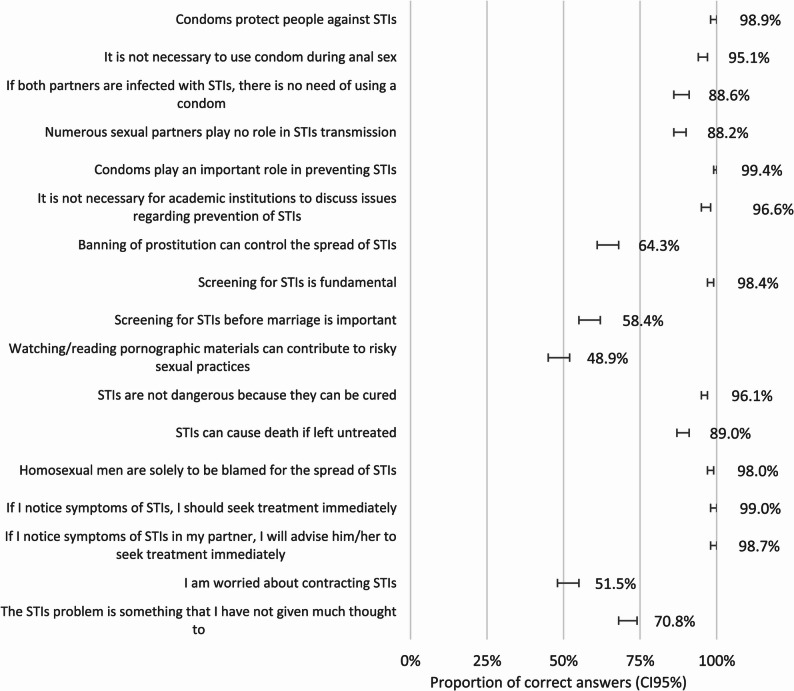
Attitudes towards STIs (proportion of correct answers)

The mean attitude score was 88.18 ± 6.01 (*n* = 790). Figure 3 presents the frequency of correct answers in each attitude-related question. Most students (98.9%) answered correctly when asked if condoms protect against STIs. The need for academic institutions to discuss STI prevention was recognised by 96.6%, and the importance of screening was recognised by 98.4%. In general, STIs were not considered a problem exclusive to men who have sex with men. Around half of the students (51.5%) admitted to being worried about contracting these infections.

When asked to rank their worries when having unprotected sex, the most voted option was acquiring HIV (49.2%). Some chose unwanted pregnancy (41.5%) as a first concern, and the least prioritised one was acquiring other STIs aside from HIV. Yet, the ones who voted for HIV as a first option were 72.8% more likely to vote for STIs, aside from HIV, as a second option.

Women were also more likely to score their attitudes higher than men (88.58 ± 5.89 vs. 87.32 ± 6.20; *p* = 0.005), and those with experience in healthcare scored higher (89.62 ± 5.72 vs. 87.76 ± 6.02; *p* < 0.001). Attitudes varied significantly across age groups (*p* < 0.001). The age range 17–21 scored the lowest (86.90 ± 5.90), and from 22 to 26 years the highest (89.29 ± 5.90). There were no significant differences between those who already had sexual intercourse and those who had not (*p* = 0.285). However, those who have had sexual intercourse exclusively with people of the opposite sex had better attitude scores (89.28 ± 5.82 vs. 87.96 ± 6.02; *p* = 0.009).

Finally, as with knowledge scores, better attitudes were observed in those who reported easier access to medication (88.34 ± 6.02 vs. 87.06 ± 5.85; *p* = 0.040) and to their assistant doctor (88.56 ± 5.94 vs. 87.62 ± 6.19; *p* = 0.045), compared to those who did not.

#### Practices

Only participants who declared having already had their first sexual intercourse were invited to answer the section on Practices (*n* = 654; 82.8%). Of those, 85 were not considered valid for scoring calculation since they did not respond to at least 80% of the questions. The proportion of missing responses in this section was 28%. In the total of 569 valid participants, the mean score of practices was 83.01 ± 8.12.

**Fig. 4:**
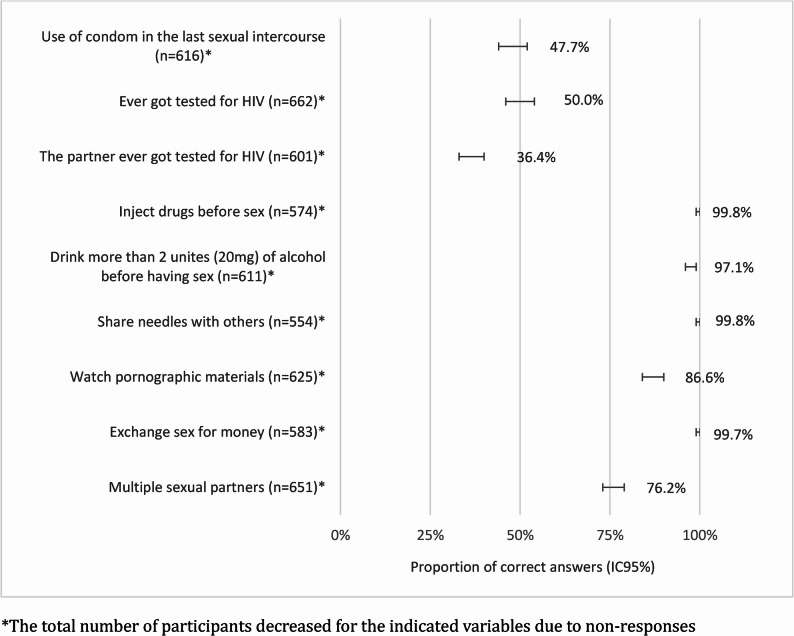
Preventive Practices and Risky Behaviours (Proportion of students engaging in a health-promotive STI behaviour) *The total number of participants decreased for the indicated variables due to non-responses

The frequency of correct answers in this section is shown in Fig. 4. All the questions allowed a non-reply option, explaining the difference in the total number of answers. The condom was used in the last sexual intercourse by 47.7%. Of the ones who didn’t use, 99.0% acknowledged the relevance of condoms in preventing STIs in the previous section. Concerning HIV testing, 51.5% declared having been tested at least once. Among the younger age group (17–21 years), 74.1% had never been tested for HIV. On the other hand, 98.0% of participants who had never been tested admitted that it is essential to screen for STIs. When asked about their partners, 39.4% reported that they had not been tested, 36.4% indicated that their partners had been tested previously, and 24.1% did not know the status of their partners’ testing. Knowing that the partner was previously tested for HIV was defined as the correct answer (Fig. 4). When asked how many partners they had in the last 12 months, 68.7% reported one, 23.8% had two or more, and 7.5% were not sexually active in this period.

As in attitudes and knowledge scores, there were differences between sex (women: 84.34 ± 7.98; men: 80.08 ± 7.67; *p* < 0.001), between age groups (27–31 years: 84.93 ± 6.65; 17–21 years: 82.06 ± 7.68; *p* = 0.017) and training in a health field (84.71 ± 7.11 vs. 82.37 ± 8.43; *p* = 0.001). Participants who had an early sexual debut had consistently lower practice scores (12–15 years: 80.94 ± 8.89, 16–21 years: 83.26 ± 7.96, ≥22 years: 85.64 ± 6.84; *p* = 0.004). Living with a romantic partner was significantly associated with better practices (mean = 85.64 ± 6.14) compared to being in a serious relationship but not living together (84.30 ± 6.70) and especially with singles (80.20 ± 9.54; *p* < 0.001). Participants who had sex with people of the same sex revealed lower scores of practices (80.18 ± 9.78 vs. 84.02 ± 7.23; *p* < 0.001). No significant association was found between practices and the ease of buying medication (*p* = 0.819) or accessing the assistant physician (*p* = 0.990).

People who were tested at least once for HIV scored higher for all KAP components (*p* < 0.001).

### Multifactorial association of Knowledge, Attitudes, Practices, and health literacy

**Fig. 5:**
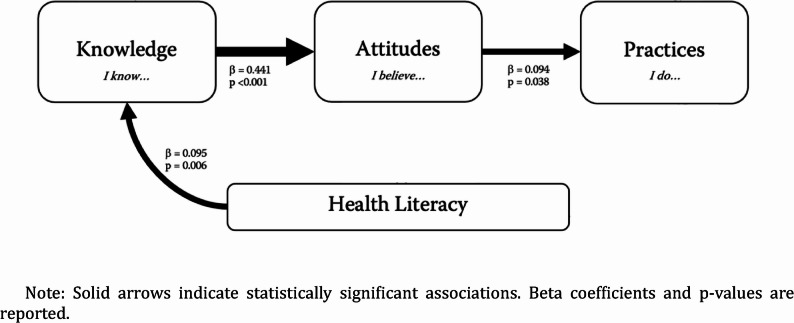
Relationship between Knowledge, Attitudes and Practices towards STIs and Health Literacy Note: Solid arrows indicate statistically significant associations. Beta coefficients and p-values are reported

Table 3 presents the adjusted linear regression models, including the covariates and the magnitude of association with the dependent variable, while Fig. 5 provides a graphical representation of the key findings.

**Table 3:** Multivariate analysis on KAP scores and covariables

	Reference	β Coefficient	95%CI	*p*-value
Model 1: *Knowledge*				
r^2^ = 0.253; F (6, 692) = 39.012; *p* < 0.001				
Sex (Female)	Male	−0.010	−1.473, 1.066	0.753
Age	-	0.003	−1.473, 1.066	0.929
Health work-related experience	No	0.462	7.977, 10.761	**< 0.001**
Health Literacy	-	0.095	0.032, 0.193	**0.006**
Easy access to medication	Difficult	0.055	−0389, 3.564	0.115
Ease of access to an assistant physician	Difficult	0.010	−1.078, 1.444	0.776
Model 2: *Attitudes*				
r^2^ = 0.189; F (7, 691) = 23.069; *p* < 0.001				
Sex (Female)	Male	0.100	0.406, 2.157	**0.004**
Age	-	0.041	−0.24, 0.091	0.253
Health work-related experience	No	− 0.122	−2.711, −0.562	**0.003**
Health Literacy	-	0.068	−0.002, 0.109	0.061
Easy access to medication	Difficult	0.010	−1.181, 1.550	0.790
Easy access to an assistant physician	Difficult	0.027	−0.536, 1.203	0.451
Knowledge	-	0.441	0.240, 0.343	**< 0.001**
Model 3: *Practices*				
r^2^ = 0.181; F (9, 511) = 12.818; *p* < 0.001				
Sex (Female)	Male	0.184	1.820, 4.708	**< 0.001**
Age	-	−0.034	−0.137, 0.058	0.429
Health work-related experience	No	0.087	−0.151, 3.309	0.074
Knowledge	-	−0.041	−0.134, 0.057	0.427
Attitudes	-	0.094	0.007, 0.259	**0.038**
Health Literacy	-	0.048	−0.036, 0.142	0.240
Relationship status (In a relationship)	Single	0.232	2.538, 5.303	**< 0.001**
Age of sexual debut		0.176	0.301, 0.811	**< 0.001**
Sexual Partner History (Same-sex/same-sex and opposite sex)	Only the opposite sex	−0.135	−1.736, −0.423	**0.001**

Model 1: multiple linear regression analysis with knowledge as the dependent variable; Model 2: multiple linear regression analysis with attitudes as the dependent variable; Model 3: multiple linear regression analysis with practice scores as the dependent variable. The results for each model are presented through r2, F-statistic (dƒ1, dƒ2) and p-values. The association between each independent variable and the outcome variable is shown through the coefficient of linear regression, 95% confidence intervals (95% CI), and* p*-values

The first model aimed to assess the determinants of higher knowledge scores (r² = 0.253; *p* < 0.001). Higher knowledge was observed in individuals with work experience in healthcare (β = 0.462; 95% CI: 7.977, 10.761; *p* < 0.001) and those with higher levels of health literacy (β = 0.095; 95% CI: 0.032, 0.193; *p* = 0.006).

The second model should identify predictors of better attitudes towards STIs (r² = 0.189; *p* < 0.001). Higher knowledge scores were significantly associated with better attitudes (t = 0.441; 95% CI: 0.240, 0.343; *p* < 0.001). A positive significant association was concluded for females (β = 0.100; 95%CI: 0.406–2.157; *p* = 0.004). However, a significant negative association was observed for those with work experience or education in healthcare (t = −0.122; 95% CI: −2.711 to −0.562; *p* = 0.003). A hierarchical multiple linear regression was performed with the same variables to investigate this unexpected result. When adjusting for knowledge scores, the contribution of work experience in healthcare changed drastically (before adjustment: β = 0.082; 95%CI: 0.055, 2.138; *p* = 0.039; after adjustment: β=−0.122; 95%CI: −2.711, −0.562; *p* = 0.003).

The variable regarding the biological sex of the previous sexual partners, which showed a significant positive association in the bivariate analysis, was excluded from these first two models due to the limited number of responses (*n* = 660).

To examine predictors of higher practice scores, a third model was conducted (r^2^ = 0.181; *p* < 0.001). Being in a relationship was associated with safer sexual practices (β = 0.232; 95%CI: 2.538, 5.303; *p* < 0.001), having had the first sexual intercourse at an older age (β = 0.176; 95%CI: 0.301, 0.811; *p* < 0.001) and being a woman (β = 0.184; 95%CI: 1.820, 4.708; *p* < 0.001). On the other hand, those who had sex with someone of the same sex, exclusively or not, revealed lower practice scores (β=−0.135; 95%CI: −1.736, −0.423; *p* = 0.001). Finally, a weak association was found between practice and attitude scores (*r* = 0.094, 95% CI: 0.007–0.259, *p* = 0.038).

## Discussion

In the present study, we assessed knowledge, attitudes, and practices related to STI acquisition and their relationship with socioeconomic characteristics, including health literacy, in Portuguese university students. Our hypothesis that knowledge, attitudes, and sexual behaviours are positively associated was partially confirmed. Individuals with higher knowledge had better attitudes towards STIs. Health literacy and knowledge of STIs exhibited a weak yet positive relationship. Yet, no significant associations were observed between practices and either knowledge or health literacy, nor between attitudes and health literacy.

### Knowledge

Self-perceived knowledge of general STIs, assessed by a single question, was adequate to profound in 50.9% of our students. Chlamydia, gonorrhoea, and syphilis were known, at least minimally, by around 90% of our students, although fewer than half reported adequate knowledge. A study conducted on Italian first-year college students found that 32% had heard about chlamydia, 45% about gonorrhoea, and 80% about syphilis [38]. A higher overall knowledge in our population may be due to our slightly older participants. However, the efforts to integrate sexual education in Portuguese schools and community-based campaigns can also explain these results [39].

Participants with a history of same-sex sexual intercourse reported higher levels of knowledge, consistent with previous research [40]. However, they scored lower on practices, aligning with the findings by Everett et al. (USA, 2014), in whom a sexual debut at a younger age, a higher number of sexual partners, and an increased odds of concurrent sexual partners were observed [20].

Those who studied or worked in healthcare had better knowledge of STIs. Still, after adjusting for knowledge, attitudes were lower and practices unchanged, suggesting that knowledge alone does not always translate into healthier sexual behaviours. Similar findings by Subotic et al. reported that, in comparison to non-medical students, the medical ones presented better STI knowledge. However, they were more sexually active, had more lifetime sexual partners, and used fewer condoms [41]. This apparent contradiction may be attributed to cognitive dissonance, psychological fatigue, desensitisation, or a misplaced sense of invulnerability. Kiragu et al. found that only 49.2% of Zambian healthcare providers used condoms in the last 12 months, with an average use in only 2.9 out of their last ten sexual intercourses. The intention to use it in the future was very low, as well as the frequency of HIV testing (only one-third), attributed mainly to their low perception of personal risk [42].

Regarding STI aetiology, there appears to be a lack of awareness about the role of fungi and parasites, as most students reported being unfamiliar with trichomoniasis, despite its prevalence [43]. In terms of transmission, kissing is not a known route for HIV, although it is for other STIs. Our participants acknowledged this route by only 27.5%. Genital Herpes, caused by Herpes simplex virus 1 and 2 (HSV-1 and HSV-2), can be transmitted by oral sex and mouth contact [44]. Syphilis can be transmitted through syphilis sores (chancres) in the lips and oropharyngeal gonorrhoea through tongue kissing [45].

Furthermore, some inconsistencies were observed in HIV knowledge; while 74% declared adequate to profound knowledge, sore throat as a symptom was rarely identified. This is concerning, as late-stage HIV made up for 52.7% of new diagnoses in 2023, which suggests an inability to recognise the early signs and symptoms [8, 46, 47]. Only half of the students had been tested in their lifetime, and the ones who did revealed better knowledge and attitudes and fewer sexual risk behaviours. Previous studies also concluded that knowledge was a relevant predictor for HIV testing [9]. Guidelines on HIV screening vary between countries. However, the European criteria have been deemed insufficient to diagnose HIV in early stages [48–50].

Other misconceptions included underestimating the impact of alcohol and substance use on STI susceptibility, despite evidence that both increase risk through behavioural and biological mechanisms [51]. Nonetheless, only 3% admitted to consuming alcohol before sexual intercourse, indicating that actual exposure in this context was low.

Sexual abstinence was thought by a significant proportion to be the most effective method to prevent STIs. However, abstinence-only programs don’t seem to decrease the risk of acquiring STIs or prevent high-risk practices in the long term [52]. Besides, 16,6% agreed that banning prostitution can help to control the transmission of STIs, whereas research in this area shows otherwise [53].

### Attitudes

Half of the students (51.5%) expressed concern about contracting an STI. Higher self-perceived knowledge confers a greater sense of control over disease prevention. In contrast, access to testing, healthcare services, and treatment could contribute to a diminished perception of personal risk [54]. Alternatively, the observed concern may reflect a lack of awareness of the potential consequences associated with STIs.

### Practices

Safer sexual practices were observed in women, individuals with a later sexual debut, and those in a romantic relationship.

Significant gaps remain, especially among younger students, who reveal lower knowledge and simultaneously higher engagement in risk behaviours, aligned with the literature [22, 24, 38, 55]. This might suggest that early sexual experiences often occur in a context of low awareness, potentially impacting future health outcomes. Although 76.4% indicated they used a condom at sexual debut, only 59.7% reported it as their usual method, and even fewer (47.7%) used it at their last sexual intercourse. According to a recent report from the World Health Organisation (WHO), around 30% of adolescents did not use a condom or a contraceptive pill at their last sexual intercourse [56]. Research also shows that adolescents who use condoms at their first sexual intercourse are 36% more likely to maintain usage even after seven years compared to those who do not [57]. Additionally, sexually active students demonstrated higher knowledge, which might suggest a learning process through experience and perhaps an early period of unsafe sex. This is concerning since STIs potentially lead to long-term consequences and reinforce the need for proper education before sexual debut [58].

Safe sexual practices depend on communication with partners. Conversely, the majority were unaware of their partner’s HIV testing status. In fact, Cegolon et al. found that merely 13.1% of university students diagnosed with an STI had disclosed this information to their partners [38]. Effective communication is crucial, since partner notification can prevent STI transmission and reinfection, yet shame, stigma, and gender-based violence often hinder this discussion [10].

Regarding multiple casual sexual partners, 23.8% of participants declared having two or more different sexual partners in the past 12 months, a proportion comparable to that observed in a Thai university-based study (27.2%) [59]. This trend increases the likelihood of unprotected sex and, consequently, the risk of contracting an STI [60].

Most of the participants were sexually active, with a mean age of sexual debut of 17.7, and 18% had sex before the age of 16. According to a 2021/2022 multinational survey, the proportion of 15-year-olds already sexually active is around 20% for boys and 15% for girls [56]. Lower practice scores were seen in students with early sexual initiation, which is consistent with the existing literature [61]. Furthermore, in our sample, females had better attitudes and practices than males. Apart from initiating earlier, boys are reported to engage in more sexual risk behaviours than girls, such as less frequent condom use and a higher number of sexual partners [62].

Sexual education interventions were proven to delay the age of sexual debut, reduce the number of sexual partners, and improve adherence to contraception and condom use [63]. It is also known that communities with structured sexual education programs have lower prevalence and incidence of HIV, higher rates of HIV testing, and more preventive behaviours towards STIs [58]. Although sexual education in Portugal has been mandatory in middle and high schools since 2009, provision has been reported as insufficient, and it is still inaccessible to more than half of young people [64]. The content of the classes focuses mainly on a biological perspective, neglecting psychological, social, and interpersonal factors [39]. In the public health centres, there is free access to family planning consultations covering contraception, healthy sexuality, unwanted pregnancy, and STIs. Nevertheless, only around one-third of teenagers have used this service [64].

### Health literacy

Most students showed insufficient health literacy, consistent with previous Portuguese studies, which reported proportions ranging from 44% to 86.8% [65–68]. No significant associations were found between HL and STI-related behaviours. This aligns with research using self-reported surveys [69], where self-report bias may reduce the accuracy of data and limit the ability to detect such associations. Moreover, a study conducted among Thai students aged 15–24 reported that those with low health literacy were 3.16 times more likely to engage in sexual risk behaviours [70]. Methodological differences may explain the discrepancy with our findings. The last study had a higher proportion of participants with low health literacy and high-risk behaviours, which likely increased statistical power and made associations easier to detect. Additionally, health literacy was assessed using a different scale, and risk behaviours were measured using only three key indicators (condom use at last intercourse, alcohol consumption before sex, and sexual activity in the previous months), creating a more apparent distinction between high- and low-risk individuals. In contrast, our study employed a set of nine questions, capturing a wider range of behaviours, which may have diluted the strength of the association. Another factor that may have contributed to the diminished association is the relative homogeneity of our sample in terms of education and access to information. Benchamas et al. concluded that individuals with higher levels of the HL dimension “application of information” were 52% more likely to have preventive sexual practices. In the same study, accessing and understanding information about sexual health had no impact on sexual behaviours [59], in line with our findings.

Our study suggests that knowledge is necessary but may be insufficient on its own. Other factors hinder individuals from adopting safer sexual practices. The application of health knowledge depends on access to healthcare, education, and income [68]. In addition to socioeconomic and educational barriers, emotional self-efficacy, cultural and social norms, and internalised stigma challenge the adoption of preventive measures and screening for STIs [71]. The college environment exposes students to alcohol and substance use, which, as discussed, lowers perceived risk of STI acquisition [72], while brain development in adolescence and early adulthood, physiological factors, and peer influence affect risk-reward and decision-making processes [73].

### Final considerations

Further research should focus on understanding the barriers to conveying sexual health information, the factors that prevent youngsters and college students from applying this information to their sexual health decisions, and the determinants that foster a risk-prevention approach in this population. In addition, the fact that knowledge of STIs is lower among first-year university students reinforces the need for further sexual education at this academic level. Developing effective sexual health education interventions is essential to curb the rise of STI cases, namely HIV, aligning with the Project 95-95−95 for the HIV pandemic by 2030 [74].

A major strength of this study is the representativeness of the sample and its focus on young people, who are both highly affected by STIs and likely to shape future public health as healthcare professionals, educators, researchers, and policymakers. Enhancing awareness in this population can inform targeted prevention strategies and contribute to long-term improvements in STI control [55].

Nevertheless, some limitations should be considered. Using an online questionnaire introduces the risk of selection bias, as students with higher health literacy may be more likely to participate in the study. Recall bias may affect the accuracy of self-reported behaviours, and the use of outside sources of information cannot be excluded. Moreover, the sections of the questionnaire, comprising Knowledge, Attitudes, and Practices, were translated from English into Portuguese but not formally validated in this language. To minimise potential bias, the translation was performed by two researchers who aimed to preserve the original meaning and ensure linguistic consistency and was submitted to a pilot test. However, the lack of cultural and linguistic validation may have affected the instrument’s reliability and comparability. Sexual behaviours and STIs are sensitive and complex topics, still encompassing stigma, discrimination, and prejudice in our society. Thus, participants may have underreported potentially undesirable behaviours and responded in a way they felt was expected of them. Moreover, the recruitment process was prone to attracting participants with higher knowledge and better practices, potentially overestimating these results compared to the target population. To minimise this bias, the questionnaire was distributed to all students at the University of Porto. The number of questions and the time required to complete it were reduced to the bare minimum, and anonymous participation was ensured. The distinction between undergraduates and postgraduates was not made. However, this was handled by adjusting for age and health-related education. On the other hand, generalising these results to all the young adults in the country may be limited because college students, with better access to education and generally higher socioeconomic status, tend to have better access to health information and services. Finally, a limitation inherent in the study’s nature is that no causal associations can be drawn.

## Conclusion

This study reveals relevant gaps between STI knowledge, attitudes, and practices among Portuguese university students. While higher knowledge was linked to better attitudes, it did not consistently translate into safer behaviours. Women, those with later sexual debut, and individuals in relationships reported safer practices, whereas early initiation and having a history of same-sex sexual intercourse were associated with riskier behaviours. Despite relatively high awareness of common STIs, misconceptions and inconsistent condom use persist, particularly among first-year students and high-risk groups. These findings highlight the urgent need for early, comprehensive sexual education that addresses not only biological facts but also social, emotional, and behavioural components. Interventions must empower students to apply knowledge effectively, dismantle stigma, and normalise testing and partner communication to curb STI rates and achieve global sexual health goals, including the WHO 95-95−95 HIV targets.

## Supplementary Information

Supplementary Material 1.

## Data Availability

The datasets used and analysed during the current study are available from the corresponding author upon reasonable request.

## Abbreviations

AIDS: Acquired immunodeficiency syndrome
CDC: Centres for disease control and prevention
CI: Confidence interval
COVID-19: Coronavirus disease 2019
EEA: European economic area
EU: European Union
HL: Health literacy
HLS-EU-Q47: European health literacy survey questionnaire
HIV: Human immunodeficiency virus
HPV: Human papillomavirus
HSV: Herpes simplex virus
IUD: Intrauterine device
KAP: Knowledge, attitudes, practices
M-POHL: Measuring population and organisational health literacy
SD: Standard deviation
STI: Sexually transmitted infection
UP: University of Porto
US: United States
WHO: World Health Organisation
