# Supplementary material for: Health literacy and risk behaviours related to sexually transmitted infections among Portuguese university students: a cross-sectional study
Source: BMC Public Health. 2026 Jan 14;26:541. doi: 10.1186/s12889-025-25904-1 (PMC12888690; doi:10.1186/s12889-025-25904-1)
Supplement: Supplementary file 1 — Supplementary Material 1. [file 12889_2025_25904_MOESM1_ESM.pdf]

Introduction and Consent

Project S4Sex: Healthy, Safe, Sensible, and Satisfying Sexuality

I am a sixth-year student in the Integrated Master's in Medicine programme. I kindly ask for your collaboration as part of my dissertation.

This questionnaire aims to assess the knowledge, skills, and attitudes of students at the University of Porto regarding sexuality. Through this survey, we hope to evaluate the factors that influence sexual satisfaction and safety, as well as key determinants in the acquisition of sexually transmitted infections (STIs).

By participating, you are contributing to progress in this field, with potential future impact on public health interventions.

The questionnaire is hosted on a Google account covered by the University of Porto's institutional licence. No personally identifiable information will be requested. The data collected will be used solely for statistical analysis within the scope of this study.

Participation is voluntary, and responses are anonymous and confidential. Your privacy and data protection are guaranteed in accordance with the European Union's General Data Protection Regulation (GDPR). You may withdraw at any time or choose not to participate, without any consequences.

Estimated completion time: 5–10 minutes.

Your contribution is very important, and I thank you in advance for your time!

For further clarification, feel free to contact me at: ***

Eligibility

1. Do you agree to participate in this survey? *

- ☐ Yes
- ☐ No → Skip to section 10 (END)

2. Are you a student at the University of Porto? *

- ☐ Yes
- ☐ No → Skip to section 10 (END)

Knowledge

3. How would you rate your knowledge of sexually transmitted infections (STIs)? *

- Scale: 1 (None) to 5 (Excellent)

4. For each of the following infections, please indicate your level of knowledge: *

- Gonorrhoea
- Syphilis

- Chlamydia
- Chancroid
- Granuloma inguinale
- Genital herpes
- HIV/AIDS
- Hepatitis B
- Trichomoniasis
  Options:
  ☐ None ☐ Minimal ☐ Basic ☐ Adequate ☐ Profound ☐ Cannot decide

5. Which organisms can cause STIs? *
   Select all that apply:
   ☐ Bacteria
   ☐ Fungi
   ☐ Viruses
   ☐ Parasites
   ☐ Mosquitoes
6. What are the transmission routes for STIs? *
   Select all that apply:
   ☐ Sexual intercourse
   ☐ Blood transfusions
   ☐ Sharing needles
   ☐ Sharing dishes/food/drinks
   ☐ Sharing clothing
   ☐ Mother-to-child transmission
   ☐ Kissing
7. Please indicate your level of agreement with the following statements: *
   1. Birth control pills reduce the risk of STIs
   2. Condoms reduce the risk of STI infection
   3. Alcohol intake increases susceptibility to STIs
   4. Drug use increases susceptibility to STIs
   5. Having multiple sexual partners increases the risk of STIs
      Options:
      ☐ Strongly disagree ☐ Disagree ☐ Neutral ☐ Agree ☐ Strongly agree
8. Symptoms of STIs include: *

   - Genital ulcers
   - Pain during urination
   - Swollen glands, fever, body aches
   - Penile discharge
   - Vaginal discharge
   - Itching around the vagina
   - Sore throat
   - Painless sores in mouth/genital area
   - It is possible to have no symptoms
     Options:
     ☐ Strongly disagree ☐ Disagree ☐ Neutral ☐ Agree ☐ Strongly agree

9. Complications of STIs include: *

   - Infertility
   - Cervical cancer
   - Fatigue

- Ectopic pregnancy
  Options:
  ☐ Strongly disagree ☐ Disagree ☐ Neutral ☐ Agree ☐ Strongly agree

Attitudes

10. Please indicate your level of agreement with the following statements: *

    - Using condoms protects against STIs
    - Condoms are not necessary during anal sex
    - If both partners are infected, condoms are unnecessary
    - Having multiple partners does not affect STI transmission
    - Condoms play an important role in STI prevention
      Options:
      ☐ Strongly disagree ☐ Disagree ☐ Neutral ☐ Agree ☐ Strongly agree

11. Please indicate your level of agreement with the following statements: *

    - I am worried about contracting an STI
    - I have never really thought about STIs as a problem
      Options:
      ☐ Strongly disagree ☐ Disagree ☐ Neutral ☐ Agree ☐ Strongly agree

12. If you have unprotected sex, what concerns you most? *
    Rank the following:

    - Contracting HIV/AIDS
    - Contracting other STIs
    - Unintended pregnancy

Sexual Practices

13. Have you ever had sexual intercourse? *

    - ☐ Yes → Continue to question 14
    - ☐ No → Skip to question 23
    - ☐ Prefer not to answer

14. Did you use a condom the last time you had sex?

    - ☐ Yes
    - ☐ No
    - ☐ Don't remember
    - ☐ Not applicable
    - ☐ Prefer not to answer

15. How many sexual partners have you had in the past 12 months?

    - ☐ 0
    - ☐ 1
    - ☐ 2
    - ☐ 3 or more
    - ☐ Prefer not to answer

16. Have you ever been tested for HIV?

    - ☐ Yes
    - ☐ No
    - ☐ Don't know
    - ☐ Prefer not to answer

17. Has your partner ever been tested for HIV? *

    - ☐ Yes
    - ☐ No
    - ☐ Don't know
    - ☐ Not applicable
    - ☐ Prefer not to answer

18. How often do you engage in the following behaviours? *

    - Inject drugs before sex
    - Drink more than 2 units of alcohol before sex
    - Share needles
    - Watch pornographic content
    - Engage in sex in exchange for money
      Options:
      ☐ Very often ☐ Often ☐ Occasionally ☐ Rarely ☐ Never ☐ Not applicable

19. At what age did you first have sexual intercourse? *
20. Did you use any contraceptive method during your first sexual intercourse?
    1. ☐ Yes
    2. ☐ No
    3. ☐ Don't know
    4. ☐ Not applicable
    5. ☐ Prefer not to answer
21. If yes, which method(s)? *
    Select all that apply:
    ☐ Condom
    ☐ Pill
    ☐ Vaginal ring
    ☐ Intrauterine device (IUD)
    ☐ Contraceptive patch
    ☐ Natural methods (temperature, calendar, cervical mucus)
    ☐ Withdrawal
    ☐ Morning-after pill
    ☐ None of the above

22. What contraceptive method do you currently use? *
    Select all that apply:
    ☐ Condom
    ☐ Pill
    ☐ Vaginal ring
    ☐ IUD
    ☐ Contraceptive patch
    ☐ Natural methods
    ☐ Withdrawal

☐ Morning-after pill
☐ None of the above

Sexual Orientation (Kinsey Scale)

23. For each question, please select the option that best describes you: *

    - Who are you attracted to?
    - Who have you had sex with?
    - Who have you had sexual fantasies about?
    - Who do you form strong emotional bonds with?
    - Who do you feel most comfortable socialising with?

    Options:
    ☐ Both men and women
    ☐ Usually people of the opposite sex
    ☐ Usually people of the same sex
    ☐ Only people of the opposite sex
    ☐ Only people of the same sex
    ☐ Not applicable

24. For each statement, indicate how you feel about the idea of having sex with: *

    - Someone of the opposite sex
    - Someone of the same sex

    Options:
    ☐ Desirable ☐ Interesting ☐ Tolerable ☐ Negative ☐ Repulsive

Health Literacy

25. For each statement, indicate how easy or difficult it is for you: *

    - Finding information about treatments for illnesses
    - Understanding how to act in medical emergencies
    - Evaluating pros and cons of treatment options
    - Following medication instructions
    - Finding information on managing mental health issues
    - Understanding the need for health screenings
    - Assessing reliability of health information on social media
    - Deciding how to protect yourself from illness

    Options:
    ☐ Very easy ☐ Easy ☐ Difficult ☐ Very difficult ☐ Don’t know

Demographics

26. **Biological sex** *
    ☐ Female
    ☐ Male
27. **Age** *
    [Open field]
28. **Faculty** *
    ☐ FADEUP (Faculty of Sports)
    ☐ FAUP (Faculty of Architecture)
    ☐ FBAUP (Faculty of Fine Arts)
    ☐ FCNAUP (Faculty of Nutrition and Food Sciences)

☐ FCUP (Faculty of Sciences)
☐ FDUP (Faculty of Law)
☐ FEP (Faculty of Economics)
☐ FEUP (Faculty of Engineering)
☐ FFUP (Faculty of Pharmacy)
☐ FLUP (Faculty of Arts and Humanities)
☐ FMDUP (Faculty of Dental Medicine)
☐ FMUP (Faculty of Medicine)
☐ FPCEUP (Faculty of Psychology and Education Sciences)
☐ ICBAS (Institute of Biomedical Sciences Abel Salazar)

29. **Which of the following statements best describes your parents' place of birth?** *
☐ Both parents were born in Portugal
☐ One parent was born in Portugal and the other in another EU Member State
☐ Both parents were born in another EU Member State
☐ One parent was born in Portugal and the other outside the EU
☐ Both parents were born outside the EU
☐ One parent was born in another EU Member State and the other outside the EU
☐ Don't know / Prefer not to answer

30. **What is your marital status?** *
☐ Single
☐ Married or in a civil partnership
☐ Separated or divorced
☐ Widowed
☐ Prefer not to answer

31. **What is your current living situation?** *
☐ Single / living alone
☐ Living together / cohabiting
☐ In a serious relationship but not living together
☐ Prefer not to answer

32. **Do you have children?** *
☐ Yes, under 15 years old
☐ Yes, over 15 years old
☐ No children
☐ Prefer not to answer

33. **What is your current employment status?** *
☐ I have a job or profession, including unpaid work for a family business or holding company (SGPS), including paid apprenticeships
☐ Full-time
☐ Part-time
☐ Unemployed
☐ Student / unpaid work experience
☐ Prefer not to answer

34. **Do you have training or have you worked in a healthcare profession (e.g. nursing, medicine, pharmacy)?** *
☐ Yes
☐ No
☐ Don't know / Prefer not to answer

35. **For each of the following, please indicate the level of difficulty:** *
Options: ☐ Very easy ☐ Easy ☐ Difficult ☐ Very difficult ☐ Don't know / Prefer not to answer

- Buying your medication to care for yourself and your family
- Accessing your GP (considering time availability, insurance/subsystem conditions, travel costs)

36. **In the past 12 months, would you say you had difficulty paying your bills at the end of the month? ***
    ☐ Most of the time
    ☐ Occasionally
    ☐ Rarely
    ☐ Never
    ☐ Don’t know / Prefer not to answer
37. **On a scale from 1 to 10, where 1 means “lowest in society” and 10 means “highest level in society,” where would you place yourself? ***
    ☐ 1 ☐ 2 ☐ 3 ☐ 4 ☐ 5 ☐ 6 ☐ 7 ☐ 8 ☐ 9 ☐ 10

End

Thank you very much for completing the questionnaire!

Please share it with your friends and help us improve knowledge in this area
